# Transcriptomic Sequencing of Airway Epithelial Cell NCI-H292 Induced by Synthetic Cationic Polypeptides

**DOI:** 10.1155/2019/3638469

**Published:** 2019-04-01

**Authors:** Ya-Ni Wang, Yu-Fei Xu, Ya-Xue Liang, Xiao-Yun Fan, Xiao-Jun Zha

**Affiliations:** ^1^Department of Geriatric Respiratory and Critical Care, The First Affiliated Hospital of Anhui Medical University, Number 218, Jixi Road, Hefei, Anhui 230022, China; ^2^Anhui Geriatric Institute, Number 218, Jixi Road, Hefei, Anhui 230022, China; ^3^Institute of Respiratory Diseases, Anhui Medical University Number 218, Jixi Road, Hefei, Anhui 230022, China; ^4^Department of Biochemistry & Molecular Biology, School of Basic Medicine, Anhui Medical University, Number 81, Meishan Road, Hefei, Anhui 230022, China

## Abstract

Eosinophil asthma is characterized by the infiltration of eosinophils to the bronchial epithelium. The toxic cationic protein released by eosinophils, mainly major basic protein (MBP), is one of the most important causative factors of epithelium damage. Poly-L-Arginine (PLA) is a kind of synthetic cationic polypeptides, which is widely used to mimic the effects of MBP on epithelial cells in vitro. However, little is known about the changes of differentially expressed genes (DEGs) and transcriptome profiles in cationic protein stimulated epithelial cells. In this study, we compared the expression of DEGs and transcriptome profiles between PLA-treated airway epithelial cells NCI-H292 and control. The results showed that there were a total of 230 DEGs, of which 86 were upregulated and 144 were downregulated. These DEGs were further analyzed using gene ontology (GO) terms and Kyoto Encyclopedia of Genes and Genomes (KEGG) pathways. The results showed that the upregulated DEGs were involved in cholesterol synthesis, protein binding, and composition of cellular membranes, mainly enriched in metabolic and biosynthesis pathways. While downregulated DEGs were implicated in cell adhesion, extracellular matrix (ECM) composition and cytoskeleton and were enriched in ECM pathway. In conclusion, our research provided the mechanism of the cationic polypeptides acting on the airway epithelial cells on the basis of transcriptomic profile, and this could be regarded as important indications in unveiling the pathologic role of natural cationic proteins in the damage to epithelial cells of asthmatics.

## 1. Introduction

Eosinophil cells are known to play a vital role in the pathophysiology of asthma, whose number in peripheral blood is relevant to the exacerbations of acute asthma. In addition, eosinophil counts are also closely related to patients' response to inhaled corticosteroids and the risk of asthma relapse [1].

Various toxic cationic proteins released by eosinophils such as major basic protein (MBP) are deemed to be essential in the etiology of asthma, especially in the pathogenesis of airway hyperresponsiveness (AHR) [2]. MBP can disrupt the epithelium in miscellaneous aspects, such as breaking the balance among ions, stimulating pulmonary edema, and directly destroying structures, as well as functions of epithelium in a charge dependent manner. Besides, cationic protein can induce apoptosis morphology changes in a concentration dependent manner in epithelial cells [3, 4]. Direct evidence suggests that cationic proteins have been found to increase in bronchial lavage fluid (BALF) in asthmatics. Meanwhile, dense deposition of MBP in the epithelium of patients who died from the status of asthmaticus is also correlated with the severity of asthma [5]. The destructive effects of cationic proteins on airway epithelium include the disruption of the epithelial structures, as well as their physiological functions. As the function of epithelial cells is impaired, airway smooth muscles are exposed, which finally result in more sensitive contractions to the agonist [6].

Airway epithelium forms the first protective barrier against environmental allergens, and their susceptibility to damages is considered to be an etiology of asthma. Considerable evidence has shown that the dysfunction of epithelial cells induced by cationic proteins has a high correlation with the hyperresponsiveness of airway smooth muscles in asthma [7]. Impaired epithelium facilitates the release of proinflammation cytokines and exposes submucosa to inhaled insults, hence resulting in higher susceptibility to antigens. Besides, structural abnormalities of epithelial cells occur before the onset of airway inflammation. In line with this, the aggregation of destroyed epithelium is observed in sputa of asthma patients. [8–11].

Poly-L-Arginine (PLA) is a kind of highly charged cationic polypeptides and shares the similar chemical molecule weight and charge with MBP. It is widely used to mimic the effects of MBP in vitro [12, 13]. However, the underlying molecular mechanism of cationic protein to the damage of epithelial cells is still unclear. In this research, we first analyzed the expression of DEGs and the transcriptomic profile, as well as the functions and related signaling pathways involved in the DEGs between the normal and the cationic polypeptides-stimulated epithelial cells. Then we chose several DEGs which were enriched in the same pathways and verified transcriptional expression of them by qRT-PCR. To our best knowledge, this is the first study to explore the mRNA expression profile in cationic polypeptides-stimulated epithelial cells by transcriptomic sequencing (RNA-Seq). This could provide a mechanistic insight into the role of cationic proteins in the pathophysiology of asthma especially in the occurrence of AHR.

## 2. Material and Methods

### 2.1. Reagents

Poly-L-Arginine (PLA) was purchased from Sigma-Aldrich (St Louis, MO, USA).

### 2.2. Experimental Design

Human lung mucoepidermoid carcinoma cell line NCI-H292 characterized by its II type alveolar epithelial cells was used in our study. It was obtained from the Shanghai Institute of Life Sciences, Chinese Academy of Sciences (Shanghai, China). Cells were cultured in 6 centimeters dishes and were divided into two groups the PLA group and the control. Each group had 3 biological replicates.

### 2.3. Cell Culture

NCI-H292 cells were cultured in 6 cm dishes with Roswell Park Memorial Institute (RPMI) 1640 Medium (Thermo Fisher Scientific, America) and supplemented with 10% fetal bovine serum (Life Technologies, Carlsbad, CA, USA) as well as 100 *μ*g/ml penicillin and 100 *μ*g/ml streptomycin (Beyotime Biotechnology, Jiangsu). Cells were incubated in a 100% humidified atmosphere with 5% CO_2_ at the temperature of 37°C.

### 2.4. Total RNA Extraction, cDNA Library Construction, and Sequencing

Total RNAs were extracted by TRIzol Reagent (Takara Bio Inc, Dalian, China) following the manufacturer's instructions. The integrity of RNA is tested by agarose gel electrophoresis, while concentrations and quality of total RNA were measured by NanoDrop ND-1000 (Thermo Fisher Scientific, Wilmington, Delaware). Afterwards, one to two micrograms of total RNA per sample were used for the construction of sequencing library with KAPA Stranded RNA-Seq Library Prep Kit (Illumina). The constructed library was then checked by Agilent 2100 Bioanalyzer and was finally quantified by qRT-PCR. According to the quantification results and the final sequencing data amount, the sequencing library mixed different samples were immersed in the sequencing process and were denatured by 0.1 M NaOH to generate single-stranded DNA. Then the single-stranded DNA was amplified using TruSeq SR Cluster Kit v3-cBot-HS (#GD-40103001, Illumina) in situ. The libraries mixed different samples were sequenced by Illumina HiSeq 4000 (service provided by Kangchen Biotech, Shanghai, China) by running 150 cycles.

### 2.5. Bioinformatics Analysis

Image analysis and base identification were performed by Solexa pipeline V1.8 (Off-Line Base Caller software, version 1.8). Sequencing quality assessment of adapter reads was performed by FastQC software [14] and cutadapt was used to remove 3′ and 5′ adapter sequences [15]. The trimmed reads were aligned with Human reference genome by Hisat2 software [16]. To estimate transcript abundance, StingTie software [17] was performed via referring to the official database annotation information. The gene and transcript expression level, FPKM value [18], and differential expressed genes and transcript expression levels were calculated using Ballgown of R software [19–21].

### 2.6. Validation of Differentially Expressed Genes

Differentially expressed genes (DEGs) were validated by quantitative real-time polymerase chain reaction (qRT-PCR). The reaction system was 20 *μ*l using SYBR® Premix Ex Taq™ II (Tli RNaseH Plus) (Takara, Dalian, China) in accordance with the manufacturer's instructions. Target genes were amplified by Bio-Rad CFX96 (Bio-Rad Laboratories, Hercules, CA). All measurements were made in triplicates. Primers of target genes were synthesized by Sangon Biotech (Shanghai, China), and the sequences of primers are showed in Table 1. The data were analyzed by comparing the Ct value method on the basis of expression of housekeeping gene GAPDH.

### 2.7. Statistical Analysis

The threshold of DEGs was set as the fold change >1.5, p-value ≤ 0.05. All results were showed as mean±standard error. Data of qRT-PCR were analyzed by SPSS version 16.0 (IBM, Armonk, NY, USA). Paired student's t-test was used for two groups comparison. It has statistically difference when p-value ≤ 0.05.

## 3. Results

### 3.1. Cluster Analysis of Differentially Expressed Genes

**Figure 1:**
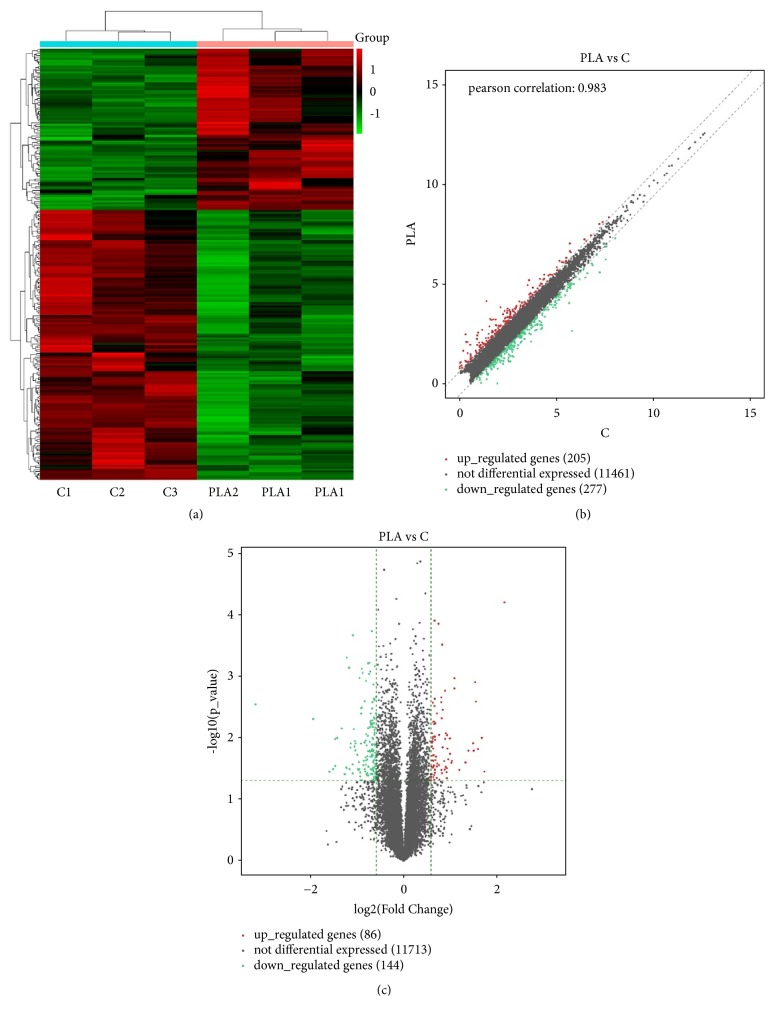
*Cluster Analysis of DEGs between PLA and Control*. (a) Hierarchical cluster of DEGs between PLA and control. Red is for upregulation and green is for downregulation. (b) Scatter Plot of DEGs. Red plots represent upregulated differentially genes; green plots are for downregulated differentially genes; grey plots represent the genes with no difference. (c) Volcano Plot of DEGs. 2 vertical green lines are upregulated (right) and downregulated (left). Red plots are upregulated differentially genes, green plots are downregulated differentially genes; grey plots represent the genes with no difference. All DEGs were selected for fold change > 1.5, and p-value ≤ 0.05.

A total of 230 genes met the defined criteria (fold change > 1.5 and p-value ≤ 0.05) and were deemed DEGs (Supplemental Excel 1). Cluster analysis of DEGs was performed by several methods as below. Hierarchical clustering has revealed the expression patterns between gene and sample. Scatter Plot has evaluated the distribution of the DEGs. Volcano plot has intuitively showed gene expression changes and corresponding statistical significance in the sample groups. 230 DEGs, including 86 upregulated and 144 downregulated genes, were exhibited (Figure 1) with the top 10 differentially expressed up- and downregulated members listed in Tables 2 and 3, respectively.

### 3.2. GO Analysis of Differentially Expressed Genes

**Figure 2:**
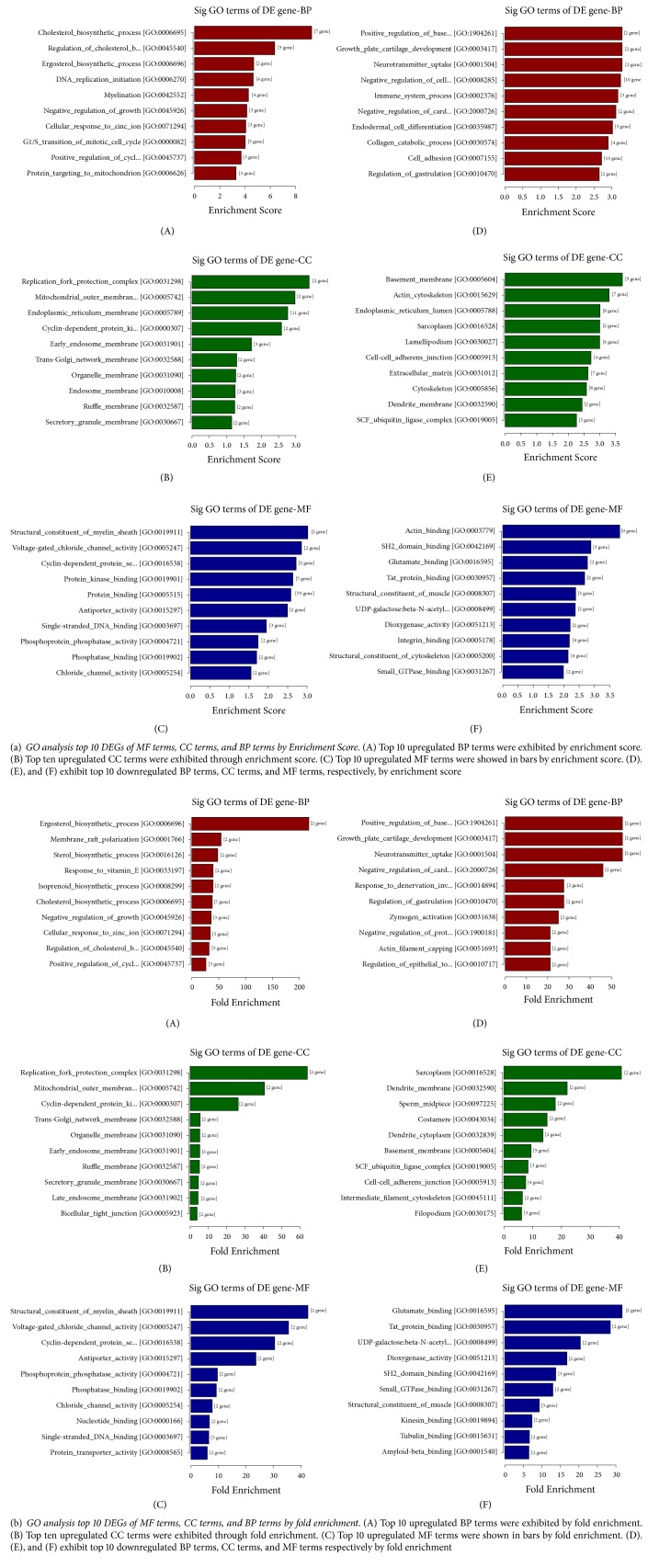
GO analyze DEGs through MF terms, CC terms, and BP terms.

In order to explore the potential biological functions of DEGs, Gene Ontology (GO) project was applied (http://www.geneontology.org). GO is a kind of gene functional classification entry that analyzes the molecular function (MF), cellular components (CC), and biological processes (BP) of DEGs [22]. It was used to clarify relationships between DEGs and certain function terms with Fisher's exact test. Each GO item corresponds to a statistical p-value which represents its statistical significance. The smaller the p-value, the greater the correlation between the DEGs and the GO terms. In other words, most of these genes have the corresponding function as GO terms described. In our study, 39 BP terms, 14 CC terms and 16 MF terms were upregulated after treated with PLA; while 79 BP terms, 38 CC terms and 26 MF terms were downregulated in contrast. Top ten significantly changed GO terms classified by BP, CC, and MF terms were listed in Figure 2.

Significantly upregulated BP, CC, and MF terms were related to the cholesterol biosynthetic process, composition of cellular membranes and protein binding respectively. In contrast, downregulated genes enriched in BP, CC, and MF terms were mainly correlated with extracellular matrix (ECM) organization, cell adhesion, basement membrane, and molecule binding, respectively.

### 3.3. KEGG Analysis of Differentially Expressed Genes

**Figure 3:**
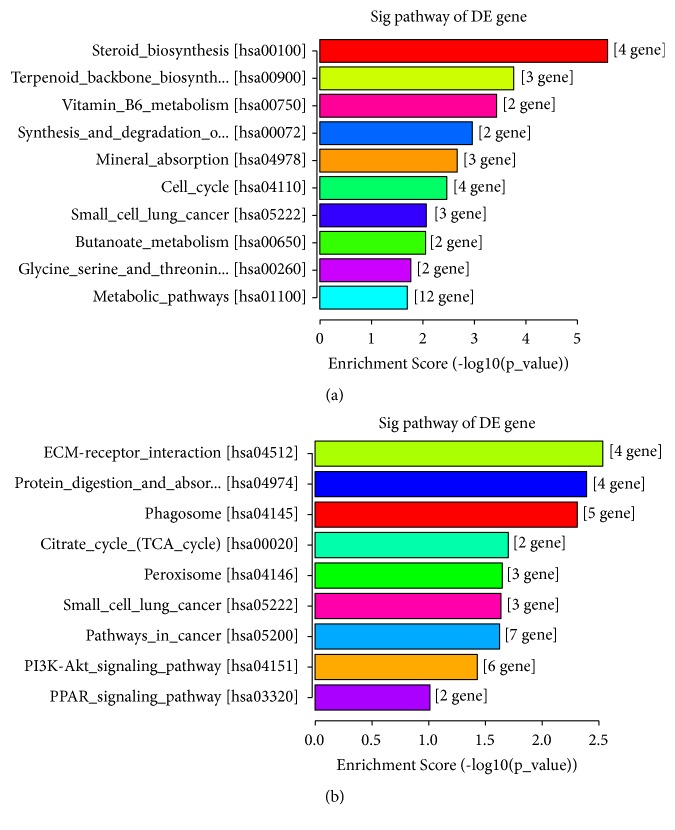
*KEGG pathway analysis*. (a) KEGG analyzed the top 10 significantly pathways of upregulated DEGs. (b) KEGG analysis of all downregulated terms. The bars are arranged from low to high according to the p-value.

Kyoto Encyclopedia of Genes and Genomes (KEGG) analysis was performed to explore the biochemical metabolic pathway and signal transduction pathways involved in these DEGs. The p-value denoted the significance of the pathway to the relative conditions (the recommend cut-off point of p-value is 0.05). As shown in the results a total of 23 pathways were significantly enriched, among which 14 pathways were upregulated and only 9 were downregulated. Upregulated DEGs were mainly related to the metabolic and biosynthesis pathways while downregulated genes were mostly abundant in ECM-receptor interaction pathway. All significantly abundant KEGG pathways and their corresponding DEGs were showed in Figure 3.

### 3.4. Validation of Differentially Expressed Genes by qRT-PCR

**Figure 4:**
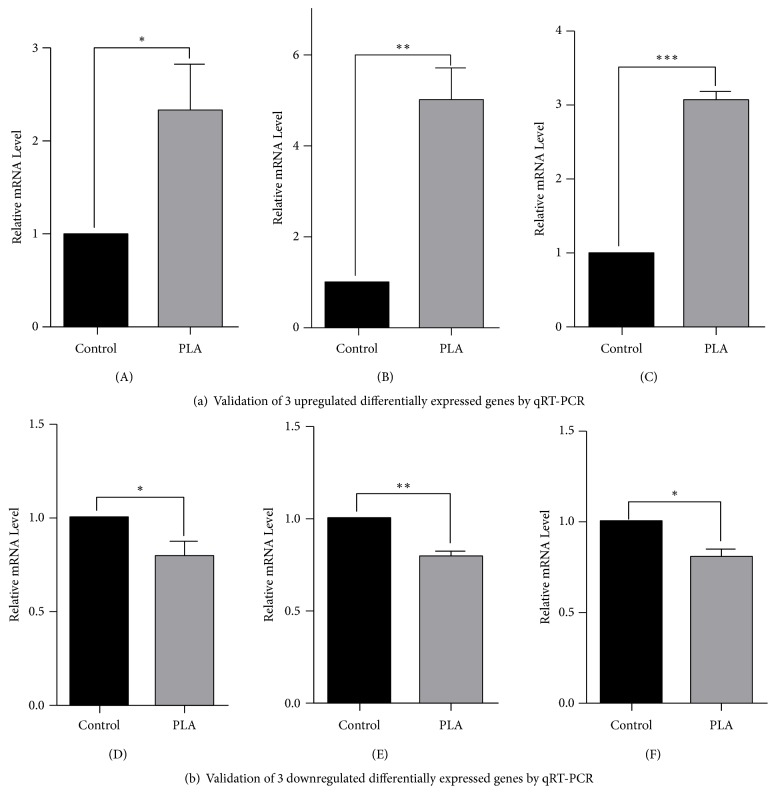
*Validation of 6 differentially expressed genes by qRT-PCR*. (A) ACAT2; (B) HMGCS1; (C) SQLE; (D) COL4A3; (E) DAG1; (F) LAMB2. Results were consistent with the RNA-Seq. **∗***p< 0.05*; **∗∗***p<0.01*; **∗∗∗***p<0.001*.

In order to validate RNA-Seq, we chose 6 DEGs by qRT-PCR: 3 upregulated DEGs, ACAT2, HMGCS1, and SQLE, which were enriched in metabolic pathway and involved in the process of cholesterol biosynthesis; 3 downregulated DEGs: COL4A3, DAG1, and LAMB2, which were enriched in ECM pathway. As the results showed, it was in accordance with RNA-Seq (Figure 4).

## 4. Discussion

Impaired structure and function of airway epithelial cells by MBP in asthma have already been demonstrated in previous studies. The impacts of MBP on cellular function include stimulating the release of histamine from basophils, attacking the pulmonary vascular systems and promoting pulmonary edema, and so on [4–6, 23]. Existence of MBP is closely related to airway hyperresponsiveness in a charge- and dose-dependent pattern. MBP could damage the integrity of epithelial membrane system, which directly leads to the exposure of subepithelial tissues like smooth muscle to exogenous pathogens. Hence, smooth muscles will present higher sensitivity and contractility to inhaled antigens in pathophysiology of asthma. Contraction of tracheal muscle is specific because it is neither induced by other natural cationic proteins secreted by eosinophils nor induced by degenerated MBP [24, 25]. Considerable researches have demonstrated that Poly-L-Arginine could be regarded as a surrogate of MBP to mimic the effect on airway epithelial cells. The morphology changes such as ultrastructure of mitochondria and membrane of epithelial cells induced by PLA have been observed [4, 26, 27]. However, up till now, little research was conducted on the changes of relevant DEGs to PLA as well as their transcriptional profile in epithelial cells. In order to compare the changes of transcriptomic profile between cationic protein-treated cells and normal, we firstly used RNA-Seq to reveal novel DEGs and transcripts.

High-throughput next-generation sequencing analyzes gene expression in an unbiased manner because it would not be affected by the selection of known genes or sequences as microarrays showed. A great number of pathogenic genes and transcripts have been found by the biotechnique. DEGs like serpin peptidase inhibitor, clade B (SERPINB2), and fibroblast growth factor binding protein 1 (FGFBP1), which were found overexpressed in biopsies of asthmatics by microarray and RNA-Seq before, were also upregulated in PLA-treated cells [28–31]. SERPINB2, also called plasminogen activator inhibitor type 2 (PAI-2), is known as an inhibitor of extracellular urokinase plasminogen activator. It plays a pivotal role in blocking the activation of plasminogen and stimulating the formation and deposition of fibrin [32]. Much fibrin was found in the sputum of asthmatics. The intense fibrin deposition could inhibit the function of surfactant that maintains the patency of airway by reducing surface tension [33, 34]. Furthermore, the expression of SERPINB2 could be greatly enhanced by allergens in asthmatics. Many researches have validated the importance of SERPINB2 in asthma and its role in inflammatory and remodeling process. Previous studies have demonstrated that SERPINB2 could be induced by IL-13, and this could be inhibited by corticosteroids in epithelial cells. In addition, expression of SERPINB2 could be found in the BALF, while the origin as well as the cause of raised SERPINB2 remains unclear. [28, 35–37]. In our study, the fold change of SERPINB2 was 3.3 in PLA-treated cells comparing to control. What is more, our research could be the first to demonstrate that PLA stimulate the expression of SERPINB2 in epithelial cells, providing probable explanations of SERPINB2 origination.

FGFBP1 is also known as FGFBP and HBP17. FGFBP1 was firstly found in human epidermoid carcinoma A431 cells, acting as a noncovalent carrier for FGF-1 or FGF-2. FGFs are related to many cellular biological processes such as morphogenesis, wound repair, inflammation, and tumor growth and invasion. In most circumstances, FGFBP1 does not express. However, abnormal expression of FGFBP1 often indicates the occurrence of carcinomas. Besides it is also upregulated in asthmatics' epithelium [30, 38–40]. FGFBP1 plays a pivotal role in the bioactivation of FGFs by binding specifically to FGF-2 in a concentration dependent manner. The combination directly leads to an increase of FGF-2 from the extracellular matrix (ECM), which ultimately invites proliferation of fibroblasts and angiogenesis. Excessive proliferation of fibroblasts contributes to airway remodeling and inflammation in asthma. Elevated FGF-2 has been found in asthmatics' bronchoalveolar lavage fluid and bronchial tissues especially in the epithelial basement membranes [39, 41, 42]. What is more, FGFBP1 also exerts an effect on the activation of MAPKs which plays a pivotal role in cell proliferation and proinflammation. The phosphorylation of ERK1/2 could be enhanced by FGFBP1. Our previous studies have shown that PLA promotes the release of IL-6 and IL-8 by activating MAPKs signaling pathway [43–45]. But the role of FGFBP1 in the activation of MAPKs induced by PLA still deserves a further research.

GO reports have provided some new insights of novel genes promoted by cationic polypeptides in asthma. As the GO reports showed, significantly enriched biological process terms were related to cholesterol biosynthetic process (also called mevalonate metabolism (MVA) synthesis), ECM organization, and cell adhesion. Abnormal cholesterol synthesis is known to be related to quantities of diseases such as obese, diabetes, and cardiac disease. It also has been reported to be associated with asthma in recent years [46]. As one of the most important components of cell membranes, cholesterol and its homeostasis are fatal for the integrity, fluidity, and signal transduction of cell membrane. Overloaded cholesterol in cell membranes contributes to the activation of Toll-like receptors and inflammasome, leading to the release of proinflammatory cytokines such as IL-1 subsequently. In animal experiments, mice with diet-induced hypercholesterolemia presented higher eosinophil counts and Th2 cytokines [47–49]. When the synthesis of cholesterol pathway is inhibited or deleted, triglyceride (TC) and cholesterol in serum decreased, leading to the resistance to hypercholesterolemia and hyperlipidemia and decreasing the related proinflammatory cell counts and cytokines. [50–52]. The high level of cholesterol in the serum also attributes to a disorder in the immune system which further abnormally mobilizes various immune cells such as macrophages, dendritic cells, and T cells, which play important roles in the pathophysiology of asthma. In line with this, hypercholesterolemic mice have exhibited a stronger TH2 immune response to antigens [53–55]. The inhibition of the cholesterol biosynthetic process by MVA inhibitors like statin in allergic asthmatics has presented a rather promising consequence. Hence, targeting MVA signaling pathway in asthma, especially for the patients with steroid resistance and obese asthma, deserves a further study [46, 56].

According to the GO reports and KEGG pathway results, PLA could upregulate HMGCS1, SQLE, and ACAT2, which were involved in cholesterol biosynthesis process and also enriched in metabolism pathway. In order to verify the consequence, we verified the expression of RNA of these three DEGs by qRT-PCR. The results in line with the RNA-Seq have provided a mechanistic insight that cationic polypeptides mainly influenced the metabolic function of the airway epithelial cells.

PLA negatively regulates the biological process of cell adhesion and varied ECM organization. We also chose three downregulated DEGs: COL4A3, LAMB2, and DAG1 for validation. These three DEGs were enriched in ECM pathway and involved in cell adhesion and ECM organization. ECM consists of an important part of pulmonary and participates in the basic cellular activity such as proliferation, adhesion, and differentiation. Recent researches have recognized that the ECM as a novel dynamic bioactive environment plays a vital role in maintaining the homeostasis of the lung [57]. The defect of the ECM is implicated in many pulmonary diseases such as chronic obstructive pulmonary diseases (COPD), idiopathic pulmonary fibrosis (IPF), and asthma. Previous researches have demonstrated that development of chronic inflammatory diseases could lead to a change of the ECM, while abnormal deposition of ECM is also related to the bronchial remodeling. Aberrant degradation of the ECM eventually results in the tissue remodeling. In our research, we found that PLA could downregulate the ECM and inhibit its organization, which could lead to a disruption of the ECM dynamic homeostasis, as well as related cellular behaviors involved cell adhesion [58–60]. Cell adhesion acts as a protective barrier for the epithelium and plays an important role against environmental allergens. In asthmatics, defective cell-cell adhesion widely exists, accompanied by downregulation of some related molecules [61, 62]. Our finding was consistent with this. Cationic polypeptides could weaken the junction between cells, increasing permeability of epithelial cells and stimulating of mucosal inflammation. From this point, we conclude that the possible mechanism of PLA in damaging epithelial cells would be the downregulation of the ECM and cell adhesion. With epithelial cells damaged, submucosal structures are exposed to inhaled allergens, and therefore airway hyperresponsiveness easily occurs.

As for CC and MF terms, membrane systems including basement membrane and organelle membranes were obviously regulated by PLA. Previous studies have shown that cationic protein could disrupt epithelial membranes in a charge dependent fashion. Under scanning electron microscope, many small pores were formed on the surface of cells after being treated with PLA, leading to the leakage of intracellular ions and the penetration of extracellular ions. Disorders of cellular ions ultimately result in lethal damages to epithelial cells such as cell death. According to GO reports, PLA diminished the calcium binding process. Increased free calcium contributed to the damage of mitochondria and cellular junction which eventually give rises to the disturbance of cell metabolism and cell death [4, 13, 63, 64]. Our findings of disrupted cellular components terms related to cell membrane and disordered molecule function terms related to ion bindings provided insights into the mechanistic basis for the observed incomplete membranes and disordered functions of epithelial cells by cationic polypeptides.

In addition, some DEGs also participate into biological processes of growth, apoptosis, macroautophagy, and so on indicating that cationic polypeptides also be related to the corresponding processes of airway epithelial cells in asthma.

KEGG have analyzed the signaling pathway which DEGs were mainly involved in. It is shown that the significantly upregulated pathways also were related to steroid biosynthesis, cell cycle, mineral absorption pathways, and so on which were involved in the regulation of cell metabolism. Downregulated pathways also participated in the peroxisome, citrate cycle pathways, and so on. The signaling pathways above might correspond to some key pathological features of asthma. Interestingly, no inflammation related pathway was exhibited in PLA-treated epithelial cells. As a surrogate of MBP, numerous studies have focused on its proinflammatory and injury effects on epithelial cells [12, 44]. However, on the basis of RNA-Seq, our research explored some novel effects of such synthetic cationic polypeptides on the airway epithelial cells. PLA appeared to have little direct proinflammatory effect on epithelial cells. But we still cannot ignore the activation of some inflammatory cytokines due to the fact that the threshold of DEGs must meet the criteria (fold change > 1.5 and P ≤ 0.05). There might still exist considerable inflammatory genes which altered slightly but could not meet the criteria. Besides, as the crosstalk between signaling pathways in the cell is complicated, we are unable to analyze the function of a certain molecule without linking it to neighboring relevant molecules. Further research should also pay attention to these molecules and their up- or downstream members.

In this study, we explored the effects of cationic synthetic polypeptides on epithelial cells by RNA-Seq on the basis of the transcription profile. However, there were still some deficiencies in the trail. Firstly, PLA was a kind of synthetic cationic polypeptides and was often used to mimic the function of natural cationic protein major basic protein (MBP) in vitro. The concentration of MBP in asthmatics varies from a few to tens of micrograms [65] and the extent of the epithelium damage by MBP could also be personalized. In vitro, the optimal concentration of cationic polypeptides on epithelium depends on previous studies and our experiments. Whether this concentration is in accordance with that in vivo still needs a further investigation. But the expression of DEGs and transcription profiles of airway epithelial cells induced by cationic polypeptides could provide some indications for the effects of natural cationic proteins on epithelial cells. Second, in this research, the cell line we chose, NCI-H292, was used to explore the expression of DEGs and transcription profiles with the following advantages: it is a kind of airway epithelial cell line and is widely used for the exploration of gene expression in airway epithelial cells in vitro; compared with other cell lines, the cell line, NCI-H292, has exhibited more different phenotypes and could represent human lung primary epithelial cells in respond to stimulus [66–68]. Despite the above advantages the cell line exhibits, more replications with more biological samples in a systemic microenvironment are also essential. Last but not least, among hundreds of DEGs we marked, it is difficult to determine the critical gene in the injury of epithelial cells. What is more, some genes pivotal in pathology of asthma which have not been found might still exist. Future researches should also strive to explore the vital genes for the damage of airway epithelial cells by cationic protein.

## 5. Conclusion

In conclusion, we have explored transcriptome profiling of human airway epithelial cells, NCI-H292, on the basis of RNA-Seq under stimulation of cationic polypeptides, PLA. Some of DEGs have been reported in asthmatics before, but many novel genes and pathways which have not been linked to asthma before were also displayed in our research. These novel findings might correspond to some key pathological features of asthma. GO reports and KEGG pathway analysis indicated that the effects of PLA on epithelial cells were mainly related to the cholesterol biosynthesis, extracellular matrix organization, and cell adhesion. All these could correspond to some key roles of natural cationic protein and its relative pathophysiological process in asthma, which could at least provide some indications for the increased permeation of cellular membranes, as well as airway hyperresponsiveness by cationic protein. In summary, our research used RNA-Seq for the first time to explore the transcriptome profiling of airway epithelial cells stimulated by cationic polypeptides, possibly serving as the basis for the study of epithelial cells under the stimulation of cationic protein in asthma in the future.

## Figures and Tables

**Table 1:** Primers applied in qRT-PCR.

Genes	Primer sequence
GAPDH	Forward 5′-TCC AAA ATC AAG TGG GGC GA-3′
	Reverser 5′-TGA TGA CCC TTT TGG CTC CC-3

ACAT2	Forward 5′-TCA ATG AAG CCT TTG CAG-3′
	Reverser 5′-CAA TAT TGA CCT TCT CTG GG-3′

HMGCS1	Forward 5′-TTG GCT TCA TGA TCT TTC AC-3′
	Reverser 5′-AAT TTA ACA TCC CCA AAG GC-3′

SQLE	Forward 5′-GGC GAG GAG GAG CGA GTC TG-3′
	Reverser 5′-ACC AAG AGG AGC ACG GAG AGC-3′

COL4A3	Forward 5′-AGCAAGGGTTGTGTCTGTAAAG-3′
	Reverse 5′-CAGAAAATCCTGGCAATCCACT-3′

DAG1	Forward 5′-CTCTCTGTGGTTATGGCTCAGT-3′
	Reverse 5′-CTGTTGGAATGGTCACTCGAAAT-3′

LAMB2	Forward 5′-CCTGGGAACTTCGACTGGG-3′
	Reverse 5′-AAGCACTTCTTTTCGTCCTGC-3′

**Table 2:** Top 10 differentially expressed upregulated genes.

Track ID	Genes Name	fold change	p value
ENSG00000241945.7_1	PWP2	4.451316597	6.27223E-05
ENSG00000197632.8_2	SERPINB2	3.312277915	0.035457486
ENSG00000134363.11_1	FST	3.186931709	0.010070294
ENSG00000178974.9_2	FBXO34	3.008371721	0.015287343
ENSG00000137440.4_1	FGFBP1	2.917532326	0.002590321
ENSG00000176624.10_1	MEX3C	2.878662293	0.00125028
ENSG00000175832.12_2	ETV4	2.823278148	0.016254865
ENSG00000144063.3_1	MALL	2.798830894	0.012302349
ENSG00000244405.7_2	ETV5	2.614272424	0.016262142
ENSG00000168461.12_2	RAB31	2.499049946	0.025288264

**Table 3:** Top 10 differentially expressed downregulated genes.

Track ID	Genes Name	fold change	p value
ENSG00000173402.11_2	DAG1	0.111305498	0.00286521
ENSG00000265590.9_2	AP000275.65	0.261138897	0.004955402
ENSG00000139112.10_2	GABARAPL1	0.332518764	0.035665775
ENSG00000173237.4_1	C11orf86	0.351447927	0.032322783
ENSG00000173227.13_2	SYT12	0.361878119	0.010471093
ENSG00000164825.3_1	DEFB1	0.362656856	0.028808651
ENSG00000167705.11_2	RILP	0.372534337	0.010070846
ENSG00000142178.7_1	SIK1	0.399425757	0.007069334
ENSG00000144476.5_2	ACKR3	0.417565995	0.039210441
ENSG00000064205.10_1	WISP2	0.426699977	0.03010091

## Data Availability

The data used to support the findings of this study are available from the corresponding author upon request.

## Supplementary Materials

Supplementary Materials Supplemental Excel 1 shows a total of 230 differentially expressed genes of which 86 were upregulated and 144 were downregulated. Table S1 lists upregulated KEGG pathways. Table S2 shows downregulated KEGG pathways.
